# SARS-CoV-2 Neutralizing Antibody Levels Post COVID-19 Vaccination Based on ELISA Method—A Small Real-World Sample Exploration

**DOI:** 10.3390/vaccines9101139

**Published:** 2021-10-06

**Authors:** Xiaoguang Li, Chao Liang, Xiumei Xiao

**Affiliations:** 1 Department of Infectious Diseases, Peking University Third Hospital, Beijing 100191, China; liang1chao1@126.com; 2 Department of Laboratory Medicine, Peking University Third Hospital, Beijing 100191, China; xxmlza@163.com

**Keywords:** SARS-CoV-2, COVID-19 vaccine, neutralizing antibody detection, enzyme-linked immunosorbent assay

## Abstract

This study investigated the detection of severe acute respiratory syndrome coronavirus 2 (SARS-CoV-2) neutralizing antibodies following inoculation with the coronavirus disease (COVID-19) vaccine. From June to July 2021, 127 participants who had completed COVID-19 vaccination (inactivated SARS-CoV-2 vaccine, 64; CoronaVac, 61; CanSino, 2) were recruited and tested using SARS-CoV-2 neutralizing antibody kits. The positive detection rate (inhibition of neutralizing antibodies ≥ 30%) was calculated and stratified according to population characteristics and inoculation time. The positive rate of neutralizing antibody was 47.22% (17/36) in men and 53.85% (49/91) in women, and 54.55% (24/44) in BMI ≥ 24 and 50.60% (42/83) in BMI < 24. Age was stratified as 20–29, 30–39, 40–49, and ≥50; positive detection rates of SARS-CoV-2 neutralizing antibodies were observed in 60.00% (24/40), 50.00% (21/42), 48.39% (15/31), and 42.86% (6/14), respectively, but with no significant difference (*x*^2^ = 1.724, *p* = 0.632). Among 127 vaccinated participants, 66 (51.97%) were positive. The positive detection rate was 63.93% (39/61) with CoronaVac and 42.19% (27/64) with the inactivated SARS-CoV-2 vaccine (significance *x*^2^ = 5.927, *p* = 0.015). Multivariate analysis revealed a significant difference in vaccination times, with average vaccination weeks in the positive and negative groups of 11.57 ± 6.48 and 17.87 ± 9.17, respectively (*t*= −4.501, *p* < 0.001). The positive neutralizing antibody rate was 100.00%, 60.00%, 58.33%, 55.56%, 43.14%, 28.57%, and 0.00% at 2–4, 5–8, 9–12, 13–16,17–20, 21–24, and >24 weeks, respectively (*x*^2^ = 18.030, *p* = 0.006). Neutralizing antibodies were detected after COVID-19 inoculation, with differences relating to inoculation timing. This study provides a reference for vaccine evaluation and follow-up immunization strengthening.

## 1. Introduction

In March 2020, the coronavirus disease (COVID-19) outbreak caused by the severe acute respiratory syndrome coronavirus 2 (SARS-CoV-2), a novel coronavirus, was recognized as a pandemic by the World Health Organization (WHO) [1]. The COVID-19 pandemic affected more than 200 countries, areas, and territories. SARS-CoV-2 belongs to the family Coronaviruses, β coronaviruses, and is a spherical single plus strand RNA virus. The virus has a phospholipid bilayer envelope with a diameter of approximately 100 nm. The nucleocapsid protein (N protein) is located inside the envelope. There are membrane proteins (M), envelope proteins (E), and spike proteins (S) in the envelope [2]. The S-protein, which consists of two functional subunits termed S1 and S2, mediates the entry of the coronavirus into host cells. The S1 subunit is responsible for binding to the host cell angiotensin converting enzyme 2 (ACE2) receptor, and the S2 subunit is responsible for cell membrane fusion. Therefore, the S protein of the coronavirus is an important target of antiviral neutralizing antibodies [3]. The neutralizing antibody blocks the binding of the virus to ACE2 mainly through interaction with the receptor binding domain (RBD) of the S protein [4].

There are three main ways for humans to obtain neutralizing antibodies: natural infection, plasma therapy, and immunization with the SARS-CoV-2 vaccine. Detection of SARS-CoV-2 neutralizing antibodies is important for the evaluation of patients’ immune status, clinical condition, prognosis, evaluation of vaccine effects, drug development, and so on.

A number of vaccines have been developed and administered to prevent COVID-19. Vaccination of the entire population is conducive to control of the epidemic. A current study [5] has shown that multiple specific neutralizing antibodies can be successfully isolated from the recovered peripheral blood of SARS-CoV-2 infected persons. The protective neutralizing antibodies and memory T cell responses after infection could maintain a stable existence for an extended period, which may be the key to avoiding secondary infection. Perhaps the protective power of the vaccine is more related to the initial immune response and less to the level of neutralizing antibodies later. Understanding the level neutralizing antibodies is also a useful exploration.

The gold standard for the serological detection of neutralizing antibodies is to use a novel coronavirus wild strain or pseudovirus detected by the cell culture method and to perform plaque reduction neutralizing assays. A high biosafety level (BSL 3 or BSL 2) is required [6]. Due to the high biosafety level requirements, clinical laboratory implementation is limited. Therefore, neutralizing antibody detection by the ELISA method would be widely carried out in clinics instead of the gold standard in the future. Sean C. Taylor [7] reported one kit for which sensitivity and specificity were 86.67% and 100%, respectively. The absorbance in the microtiter plate reader at 450 nm was read. The inhibition was calculated, and 30% is the criterion for positive neutralization antibody. This kind of kit was used in this study. Therefore, this study did not adopt quantitative indicators, but rather qualitative indicators according to the kit instructions, and the positive detection rate was taken as the main research indicator.

The purpose of this study was to detect the level of SARS-CoV-2 neutralizing antibodies after COVID-19 vaccination based on a small sample in the real world, to provide data support for epidemic prevention and control and to provide a reference for the subsequent need for re-injection of COVID-19 vaccine to strengthen immunization.

## 2. Objects and Methods

### 2.1. Research Participants

The study recruited medical staff and related personnel from the Peking University Third Hospital from June to July 2021. The inclusion criteria were persons who had received the COVID-19 vaccine and who had signed an informed consent form. Those who could not cooperate with the completion of blood collection and visit were excluded. A total of 127 subjects who received the COVID-19 vaccine were selected for this study. Among the 127 subjects, 61 cases received the two doses of inactivated SARS-CoV-2 vaccine developed by Beijing Sinovac Science & Technology Co., Beijing, China, LTD (abbreviation CoronaVac), 64 cases received the two doses of inactivated SARS-CoV-2 vaccine (Vero cells) from the Beijing Institute of Biological Products/Sinopharm, Beijing, China, (abbreviation inactivated SARS-CoV-2 vaccine), and two cases were vaccinated with the one dose recombinant adenovirus type 5 vector vaccine manufactured by CanSino Biological Inc., Beijing Institute of Biotechnology (abbreviation CanSino). This study was approved by the Medical Science Research Ethics Committee of Peking University Third Hospital (No. 2021- 129-02 2021(129-02)). All participants provided written informed consent.

### 2.2. Research Methods

A 4 mL whole blood specimen was collected from each participant and centrifuged at 3000 RPM for 10 min. Then, 2 ml of serum was separated and stored at −20 °C for detection within two months.

### 2.3. Instruments

Instruments used in this study included a high-speed refrigerated centrifuge (Eppendorf 5810R, Hamburg, Germany), microplate instrument (Thermo Fisher Scientific, Waltham, MA, USA, model MK3), and a waterproof constant temperature incubator (Shanghai Fuma Experimental Equipment Co., Ltd., Shanghai, China, model X-908B-1).

### 2.4. Reagents and Methods

The cPass SARS-CoV-2 Neutralization Antibody Detection kit (ELISA) manufactured by GenScript Biotechnology Co., Ltd., Nanjing, China, was used, following the manufacturer’s instructions.

### 2.5. Assay Principle

The kit is a blocking ELISA detection tool, which mimics the virus neutralization process. The kit contains two key components: the horseradish peroxidase (HRP) conjugated recombinant SARS-CoV-2 RBD fragment (HRP-RBD) and the human ACE2 receptor protein (hACE2). The protein–protein interaction between HRP-RBD and hACE2 can be blocked by neutralizing antibodies against SARS-CoV-2 RBD.

### 2.6. Reagent Preparation

HRP-conjugated RBD was diluted with an HRP dilution buffer at a volume ratio of 1:1000. The 1× wash solution was prepared by diluting the 20× wash solution with deionized or distilled water at a volume ratio of 1:19. Then, for the sample and control dilution, the test sample and positive and negative controls were diluted with sample dilution buffer at a volume ratio of 1:9.

### 2.7. Capture Plate Preparation

All positive and negative controls were prepared in duplicate. The strips were counted, numbered, and installed, making sure the strips were tightly snapped into the plate frame.

### 2.8. Test Procedure

In separate tubes, the positive control/negative control/samples were mixed with the diluted HRP-RBD solution at a volume ratio of 1:1, and incubated at 37 °C for 30 min. Then, 100 μL each of the positive control, the negative control, and the sample mixture was added to corresponding wells. The plate was covered with a plate sealer and incubated at 37 °C for 15 min. The plate sealer was removed, and the plate was washed four times with 260 μL of the 1× wash solution. The plate was placed on a paper towel to remove residual liquid in the wells after washing. Subsequently, 100 μL of TMB solution was added to each well, and the plate was incubated in the dark at 20–25 °C for 15 min. Finally, 50 μL of Stop Solution was added to each well to quench the reaction, and the absorbance in the microtiter plate reader at 450 nm was read immediately.

### 2.9. Interpretation of Results

Inhibition was calculated as (1 − optical density [OD] value of sample/OD value of negative control) × 100%. An inhibition rate of ≥30% was considered positive for SARS-CoV-2 neutralizing antibodies, while <30% was considered negative.

### 2.10. Statistical Methods

Statistical analysis was performed using SPSS version 17 software (SPSS, Inc., Chicago, IL, USA). The measurement data are expressed as the mean ± standard deviation. The counting data were expressed as frequency and percentage and divided into positive and negative groups according to the detection of SARS-CoV-2 neutralizing antibody. For comparison between groups, a single-factor analysis was first used. An independent sample *t*-test was used for quantitative data, and the *x*^2^ test was used for qualitative data. Second, binary logistic regression analysis was performed. Statistical significance was set at *p* < 0.05.

## 3. Results

### 3.1. Basic Information

Basic information on the 127 participants, such as demographic data, is shown in Table 1. There were 36 men and 91 women, for a male-to-female ratio of 1:2.5. The average age was 36.50 ± 10.61 years and ranged from 22 to 73 years. A total of 97 participants (76.38%) were medical personnel, and 30 (23.62%) were other related personnel. None of the patients had a history of or exposure to COVID-19. All cases had a green health code and were negative for SARS-CoV-2 nucleic acid tests. The demographic characteristics between CoronaVac and Inactivated SARS-CoV-2 vaccine recipients showed no significant difference (*p* > 0.05) in Supplementary Table S1. Therefore, the demographic characteristics baseline levels are comparable between the CoronaVac and inactivated SARS-CoV-2 vaccine.

### 3.2. Single Factor Analysis of Demographic Characteristics

Among the 127 subjects, 66 were positive and 61 were negative, for a positive detection rate of 51.97%. The positive rate of neutralizing antibody was 47.22% (17/36) in men and 53.85% (49/91) in women, and 54.55% (24/44) in BMI ≥ 24 and 50.60% (42/83) in BMI < 24.There were no significant differences in sex, age, height, weight, and body mass index (BMI) between the positive and negative SARS-CoV-2 neutralizing antibody groups (*p* > 0.05), as shown in Table 2.

Age was further stratified as 20–29 years, 30–39 years, 40–49 years, and ≥50 years, and positive detection rates of SARS-CoV-2 neutralizing antibodies were observed in 60.00% (24/40), 50.00% (21/42), 48.39% (15/31), and 42.86% (6/14), respectively, with no significant difference (*x*^2^ = 1.724, *p* = 0.632), as shown in Table 2.

### 3.3. Single Factor Analysis of Vaccination Status

Among the 127 subjects, seven were vaccinated in 2020, and 120 completed vaccinations in 2021. In 2020, the vaccine was in a phase III clinical trial, with five participants inoculated in August, one in November, and one in December. All seven cases tested negative for neutralizing antibodies. In 2021, a variety of the vaccines were marketed in China, and 66 out of 120 cases were positive, for a positive rate of 55.00% (66/120).

In this study, there are three kinds of vaccines involved (inactivated SARS-CoV-2 vaccine, 64; CoronaVac, 61; CanSino, 2). Among 127 vaccinated participants, 66 (51.97%) were positive. The positive detection rate was 63.93% (39/61) with CoronaVac and 42.19% (27/64) with inactivated SARS-CoV-2 vaccine (significance *x*^2^ = 5.927, *p* = 0.015), as shown in Table 3. The neutralizing antibody test was negative in the two cases vaccinated with CanSino; time after inoculation was 27 weeks in one case and five weeks in the other. The time (weeks) from completion of vaccination with different vaccine type is shown in Supplementary Table S2. There was a significant difference in vaccination times, with average vaccination weeks in CoronaVac and inactivated SARS-CoV-2 vaccine groups of 12.30 ± 10.34 and 16.72 ± 5.26, respectively (*t* = −2.996, *p* = 0.004).

Further analysis of neutralization antibody positive and negative groups according to the time (weeks) from completion of vaccination showed a significant difference (*t* = −4.501, *p* < 0.001). The average vaccination weeks of the positive group was 11.57 ± 6.48 and that of the negative group was 17.87 ± 9.17. The positive rate of neutralizing antibody was 92.31%, 60.00%, 58.33%, 55.56%, 43.14%, 28.57%, and 0.00% at 2–4, 5–8, 9–12, 13–16, 17–20, 21–24, and more than 24 weeks, respectively, and the difference was significant (*x*^2^ = 18.030, *p* = 0.006), as shown in Table 3.

### 3.4. Logistic Regression Multivariate Analysis

Sex, age, height, weight, type of vaccine, and number of weeks of vaccination were analyzed by multivariate analysis, and binary logistic regression analysis was performed. After variable selection, the results showed that the number of weeks of vaccination was statistically significant (*p* = 0.001, odds ratio [OR] = 0.613), as shown in Table 4. Vaccine type was not statistically significant (*p* = 0.413, OR = 1.438). There were no statistically significant differences in age groups (*p* > 0.05). The correlation coefficient between the type of vaccine and the number of weeks of vaccination was 0.357.

## 4. Discussion

This study aimed to detect the level of SARS-CoV-2 neutralizing antibodies after COVID-19 vaccination based on a small real-world sample. It provides a reference that can be used for public health decisions, assessing the need for supplementary vaccination, and determining the time interval between vaccinations. Furthermore, according to this ELISA kit, this study found that the overall positive antibody result among 127 vaccinated participants was 51.97%. According to single factor analysis, there were significant differences in positive results based on the vaccination time and type of vaccination, which could be used to facilitate optimal vaccination results. However, according to multivariate analysis, only the timing of vaccination was significant.

Serum immunological testing is a common method for detecting COVID-19. Serological antibody tests for COVID-19 can be performed using different techniques, including enzyme-linked immunosorbent assay (ELISA) chemiluminescence immunoassay (CLIA), indirect immunofluorescence test (IIFT), lateral flow immunoassay, immunochromatography, Western blot-based assays, and virus neutralization assays [8]. These techniques allow the routine detection of several classes of antibodies, such as IgM, IgG, and IgA [9,10,11,12]. Regarding the urgent need for accurate, easy, and rapid methods for screening asymptomatic carriers, a meta-analysis showed the sensitivity of these kits for both IgM and IgG tests was between 72.7% and 100%, while specificity ranged between 98.7% and 100% [13].

To understand immunity after natural infection or vaccination, a functional analysis of the elicited antibody responses, such as avidity for the most immunogenic viral antigens and virus neutralizing activity, is of utmost importance [14,15,16]. The gold standard for the serological detection of neutralizing antibodies is to use a novel coronavirus wild strain or pseudovirus detected by the cell culture method and to perform plaque reduction neutralizing assays, which required the high biosafety level [7]. At present, many companies have solved this bottleneck by successfully developing immunological SARS-CoV-2 antibody detection kits [15,16].

The GenScript kit was used in this study. The ACE2 package on the 96-well plate, using ELISA method, detects the inhibition rate of the virus S protein RBD binding with ACE2. The inhibition rate was used to evaluate the serum neutralizing antibody. ELISA is a classic method in clinical laboratories, which has the characteristics of low cost, high-throughput, and is more suitable for large-scale population detection. Moreover, microplate standing equipment in clinical testing institutions have a wide application basis and can satisfy the needs of common laboratories to carry out population testing [16].

The significance of neutralizing antibody detection has been recognized gradually. In this study, single factor analysis identified two significant factors: vaccine manufacturer and vaccination time. The positive detection rate of neutralizing antibodies against the CoronaVac vaccine was 63.93%, and that of the inactivated SARS-CoV-2 vaccine was 42.19% (*p* < 0.05). The inoculation time was 11.57 ± 6.48 weeks in the positive SARS-CoV-2 neutralizing antibody group and 17.87 ± 9.17 weeks in the negative group, with significant difference (*p* < 0.05). In this study, with inoculation time of 2–4 weeks, the positive rate of neutralizing antibodies was 100%. Meanwhile, six participants had been inoculated for more than 24 weeks, and all were negative for neutralizing antibodies. Multivariate logistic analysis showed that only the time of inoculation was statistically significant. Therefore, the inoculation time was a very important factor. At the time, most of the initial vaccinations were delivered to medical workers and other people with high occupational exposure, mostly in clinical trial phase vaccinations. The time of inoculation exceeded half a year and was close to one year. Considering that the protective power of vaccines could decrease over time, it is necessary for these people to be revaccinated to strengthen their immunity. Johan Normark [17] already reported strengthen immunity that mRNA vaccines (here in the form of mRNA1273) may be useful for vaccination strategies in which a third dose is to be administered to persons who have previously received two doses of ChAdOx1 nCoV-19. Neutralizing antibody levels also need to be monitored to assess the third dose of a vaccine.

A previous meta-analysis including eleven studies of the COVID-19 vaccine and neutralizing antibodies found that all candidate vaccines induced high levels of neutralizing antibodies—Bbibp-corv, AZD1222, BNT162b2, New Crown COVID-19, and Sputnik V—had a significant impact on the level of neutralizing antibodies (SMD > 1.3) [18]. In a real-world study of the CoronaVac vaccine, among the 10.2 million people who received two doses of the vaccine, the estimated protection against COVID-19, hospitalization for COVID-19, hospitalization in an intensive care unit, and related deaths were 65.9%, 87.5%, 90.3%, and 86.3%, respectively [19].

As for the relationship between age and the level of neutralizing antibody, this study found that the positive rates of neutralizing antibody showed a gradual downward trend related to increased age, but there was no significant difference (*p* > 0.05). In another report, older and middle-aged patients had significantly higher plasma neutralizing antibody titers than those of young patients. The titers of neutralizing antibodies were positively correlated with plasma C-reactive protein levels and negatively correlated with the lymphocyte counts of patients at the time of admission [18]. More individuals should be studied to estimate whether the vaccine is less protective in older people than in younger people.

The relationship between disease severity and neutralizing antibodies showed that the critical cases had higher anti-RBD IgG than that in the mild/moderate cases, but anti-RBD IgM OD was not correlated with disease severity. Additionally, confirmatory microneutralization and 90% plaque reduction neutralization tests were positive four weeks after disease onset, while there was no detectable cross-reactivity in the control group [20].

In a study of the time interval between two dose vaccinations, Oxford University reported that overall vaccine efficacy more than 14 days after the second dose was 66.7% (95% CI 57.4–74.0). Exploratory analyses showed that vaccine efficacy after a single standard dose of vaccine from day 22 to day 90 after vaccination was 76.0% (59.3–85.9). The modelling analysis indicated that protection did not wane during the initial three-month period. In the participants who received two standard doses, after the second dose, efficacy was higher in those with a longer prime-boost interval (vaccine efficacy 81.3% [95% CI 60.3–91.2] at ≥12 weeks) than in those with a short interval (vaccine efficacy 55.1% [33.0–69.9] at <6 weeks) [21].

At present, COVID-19 still need to be monitored. The continued fluctuation of the epidemic is closely related to the mutation of the virus. Worldwide, SARS-CoV-2 has evolved a variety of mutant strains, mainly including alpha mutant, beta mutant, gamma mutant, Delta mutant, etc. [22]. The emergence of variants of SARS-CoV-2 B.1.1.7 in the United Kingdom and B.1.351 in South Africa has aroused concern that these variants may escape immunity resulting from either previous infection or vaccination. There is a need to study the susceptibility of SARS-CoV-2 variants to neutralization. A previous study [23] evaluated the resistance of pseudovirus to neutralization by using a convalescent serum obtained from 34 patients five months after infection with COVID-19 and serum from 50 participants obtained two to three weeks after receipt of the second dose of inactivated-virus vaccines—BBIBP-CorV (Sinopharm) or CoronaVac (Sinovac). They found that B.1.1.7 showed little resistance to the neutralizing activity of convalescent or vaccine serum. Most of the vaccine serum samples that were tested lost neutralizing activity, a finding that was consistent with the results of other recent studies of neutralization by convalescent serum or serum obtained from recipients of messenger RNA or BBIBP-CorV vaccines [24,25,26].

The other question concerns the cross-reactivity of the neutralizing antibodies. It should be defined whether previous infections with a certain strain of coronaviruses could protect individuals against future strains. Accordingly, a recent serological assessment of 175 COVID-19 recovered patients with mild symptoms using RBD, S1, and S2 antigens of SARS-CoV-2 demonstrated that SARS-CoV-2 neutralizing antibodies had no cross-reactivity with SARS-CoV virus [27].

At present, the cases of COVID-19 in China are mainly imported cases and sporadic local cases. In this study, none of the 127 subjects included had a breakthrough infection in the current follow-up. There were no adverse events or deaths in this study. The magazine Cell [28] reported that in 113 COVID-19 patients, COVID-19-neutralizing antibodies predicted disease severity and survival. However, in clinical vaccine trials, high levels of neutralizing antibodies in vaccinated populations predicted a reduced risk of ICU admission, severe illness, and death [29]. Therefore, monitoring neutralizing antibody levels has implications for both COVID-19 patients and vaccinators.

This study was limited by method and subjects. ELISA has many limitations. While RBD specific neutralizing antibody is the most important, there are other targeting antibodies, which were not evaluated in this study. Hence, this study did not provide the actual overall virus neutralization response. Moreover, ELISA-based detection of antibodies might provide some information about antibody binding affinity, but it does not necessarily mean that these antibodies can also neutralize the virus. The ELISA method adopted in this study may be false positives and false negatives.

There was a small sample size and bias in the structure of the participants. Medical personnel and women were mainly recruited, and the vaccination time was relatively concentrated. In the context of strict epidemic prevention and control in Beijing, it is difficult to directly observe whether vaccination is administered and to compare it with prognostic factors, such as the number of infected people, the proportion of hospitalized patients, the proportion of severe cases, and the mortality rate. Vaccine protection can only be assessed indirectly by detecting neutralizing antibody levels, which only provide a limited reference for immune status.

In summary, this study aimed to explore SARS-CoV-2 neutralizing antibody levels after vaccination based on ELISA with a small real-world sample. With prolonged inoculation, neutralizing antibody levels could be reduced. The neutralizing antibody test could be used to assess the protection of vaccination, but a positive result does not indicate absolute protection, and a negative result does not necessarily indicate non-protection. This was only used as a reference. It provides a reference for public health decisions, whether to advise supplementary vaccination, and the optimal time interval between doses to strengthen the immune system.

## Figures and Tables

**Table 1:** Demographic characteristics of 127 COVID-19 vaccine recipients.

Item		Total *N*	Mean ± SD/%
Sex	Male	36	28.35%
	Female	91	71.65%
Age (years)			36.50 ± 10.61
	20–29	40	31.5%
	30–39	42	33.1
	40–49	31	24.4
	≥50	14	11.0%
Height (cm)			165.59 ± 7.20
Weight (kg)			62.88 ± 11.00
BMI (kg/m^2^)			22.85 ± 3.15
	≥24	44	34.65%
	18.5 < BMI < 24	76	59.84%
	≤18.5	7	5.51%

127 participants who had completed COVID-19 vaccination (inactivated SARS-CoV-2 vaccine, 64; CoronaVac, 61; CanSino, 2). SD: standard deviation; BMI: body mass index.

**Table 2:** The results of SARS-CoV-2 neutralization antibodies in 127 cases classified by demographic characteristics.

Item	Positive Group	Negative Group	*t*/*x*^2^	*p* Value
*N* = 127	66	61		
Sex Male	17 (47.22%)	19 (52.78%)	0.453	0.501
Female	49 (53.85%)	42 (46.15%)		
Age (years)	35.06 ± 10.28	38.05 ± 10.83	−1.595	0.113
20–29	24 (60.00%)	16 (40.00%)	1.724	0.632
30–39	21 (50.00%)	21 (50.00%)		
40–49	15 (48.39%)	16 (51.61%)		
≥50	6 (42.86%)	8 (57.14%)		
Height (cm)	166.05 ± 7.43	165.11 ± 6.98	0.732	0.465
Weight (kg)	63.26 ± 11.78	62.48 ± 10.16	0.393	0.695
BMI (kg/m^2^)	22.83 ± 3.09	22.88 ± 3.25	−0.090	0.929
≥24	24 (54.55%)	20 (45.45%)	0.179	0.672
<24	42 (50.60%)	41 (49.40%)		

127 participants who had completed COVID-19 vaccination (inactivated SARS-CoV-2 vaccine, 64; CoronaVac, 61; CanSino, 2). BMI: body mass index.

**Table 3:** Results of SARS-CoV-2 neutralizing antibodies in 127 cases by vaccination status.

Item	*N*	Positive Group	Negative Group	*t*/*x*^2^	*p* Value
	127	66	61		
Time of vaccination completed (weeks)		11.57 ± 6.48	17.87 ± 9.17	−4.501	<0.001
					
2–4	12	12 (100.00%)	0 (0.00%)	18.030	0.006
5–8	30	18 (60.00%)	12 (40.00%)		
9–12	12	7 (58.33%)	5 (41.67%)		
13–16	9	5 (55.56%)	4 (44.44%)		
17–20	51	22 (43.14%)	29 (56.86%)		
21–24	7	2 (28.57%)	5 (71.43%)		
>24	6	0 (0.00%)	6 (100.00%)		
Vaccine manufacturers *					
CoronaVac	61	39 (63.93%)	22 (36.07%)	5.927	0.015
inactivated SARS-CoV-2 vaccine	64	27 (42.19%)	37 (57.81%)		

* 127 participants who had completed COVID-19 vaccination (inactivated SARS-CoV-2 vaccine, 64; CoronaVac, 61; CanSino, 2).

**Table 4:** Results of multivariate analysis of SARS-CoV-2 neutralizing antibodies by logistic regression model.

Item	Regression Coefficient	Standard Error	Wald *x*^2^	*p* Value	OR	95% CI
Sex	−0.764	0.621	1.513	0.219	0.466	(0.138, 1.573)
Age	−0.029	0.021	1.946	0.163	0.972	(0.933, 1.012)
Height	0.045	0.046	0.966	0.326	1.046	(0.956, 1.144)
Weight	0.005	0.025	0.040	0.842	1.005	(0.957, 1.055)
Vaccine manufacturers	0.463	0.435	1.136	0.287	1.589	(0.678, 3.727)
Time of vaccination completed (weeks)	−0.109	0.032	11.569	0.001	0.897	(0.842, 0.955)

127 participants who had completed COVID-19 vaccination (inactivated SARS-CoV-2 vaccine, 64; CoronaVac, 61; CanSino, 2).

## Data Availability

The data supporting the findings of this study are available upon reasonable request from the authors.

## Supplementary Materials

The following are available online at www.mdpi.com/article/10.3390/vaccines9101139/s1, Table S1: The demographic characteristics between CoronaVac and Inactivated SARS-CoV-2 vaccine group, Table S2: Time of vaccination completed (weeks) between CoronaVac and Inactivated SARS-CoV-2 vaccine group.
