# Supplementary material for: SARS-CoV-2 Neutralizing Antibody Levels Post COVID-19 Vaccination Based on ELISA Method—A Small Real-World Sample Exploration
Source: Vaccines (Basel). 2021 Oct 6;9(10):1139. doi: 10.3390/vaccines9101139 (PMC8541171; doi:10.3390/vaccines9101139)
Supplement: Supplementary file 1 [file vaccines-09-01139-s001.zip › vaccines-1365883-supplementary.pdf]

Table S1 The demographic characteristics between CoronaVac and Inactivated SARS-CoV-2 vaccine group

Item		CoronaVac N=61	Mean±SD / %	Inactivated SARS-CoV-2 vaccine N=64	Mean±SD / %	t / x^2	*P* value
Sex	Male	19	31.15%	16	25.00%	0.585	0.444
	Female	42	68.85%	48	75.00%		
Age (years)		36.43±11.20		36.59±9.99		-0.088	0.930
	20–29	20	32.79%	19	29.69%	2.012	0.570
	30–39	17	27.87%	25	39.06%		
	40–49	17	27.87%	13	20.31%		
	⩾50	7	11.48%	7	10.94%		
Height (cm)		166.19±7.45		165.01±6.85		0.896	0.372
Weight (kg)		63.16±11.30		62.76±10.36		0.201	0.841
BMI （kg/m^2）		22.76±2.89		23.01±3.30		-0.452	0.652
	⩾24	18	29.51%	25	39.06%	1.263	0.261
	18.5 < BMI < 24	42	68.85%	34	53.13%		
	⩽18.5	1	1.64%	5	7.81%		

127 participants who had completed COVID-19 vaccination (inactivated SARS-CoV-2 vaccine, 64; CoronaVac, 61; CanSino, 2)

Table S2 Time of vaccination completed (weeks) between CoronaVac and Inactivated SARS-CoV-2 vaccine group

	CoronaVac		inactivated SARS-CoV-2 vaccine		CanSino	
	N=61	positive n(%)	N=64	positive n (%)	N=2	positive n (%)
Time of vaccination completed (weeks)		12.30±10.34*		16.72±5.26*		
2-4	11	11(91.67%)	1	1(100.00%)		
5-8	20	14(70.00%)	9	4(44.44%)	1	0
9-12	8	4(50.00%)	4	3(75.00%)		
13-16	7	3(42.86%)	2	2(100.00%)		
17-20	9	7(77.78%)	42	15(35.71%)		
21-24	1	0(0.00%)	6	2(33.33%)		
>24	5	0(0.00%)			1	0

127 participants who had completed COVID-19 vaccination (inactivated SARS-CoV-2 vaccine, 64; CoronaVac, 61; CanSino, 2)

* significant difference in vaccination times, with average vaccination weeks in CoronaVac and inactivated SARS-CoV-2 vaccine groups of 12.30±10.34 and 16.72±5.26, respectively (t =-2.996, P=0.004).
